# Guest editorial: Overview, implications, and directions of twin design research exploring neurodevelopmental conditions

**DOI:** 10.1002/jcv2.12121

**Published:** 2022-11-17

**Authors:** Lynnea Myers, Sven Bölte

**Affiliations:** ^1^ Division of Clinical Pediatrics Department of Women's and Children's Health Karolinska Institutet Stockholm Sweden; ^2^ Precision Population Science Lab Mayo Clinic Rochester Minnesota USA; ^3^ Department of Nursing Gustavus Adolphus College St. Peter Minnesota USA; ^4^ Department of Women's and Children's Health Centre for Psychiatry Research Karolinska Institutet Center of Neurodevelopmental Disorder (KIND) Karolinska Institutet & Stockholm Health Care Services Region Stockholm Stockholm Sweden; ^5^ Child and Adolescent Psychiatry Stockholm Health Care Services Region Stockholm Stockholm Sweden; ^6^ Autism Research Group Curtin School of Allied Health Curtin University Perth Western Australia Australia

We are honored that our publication, “Behavioral and biological divergence in monozygotic twin pairs discordant for autism phenotypes: A systematic review” was chosen for the Best Paper Award 2022 in JCPP *Advances.* In the awarded paper, we examine published evidence on non‐shared environment effects in individuals diagnosed with autism spectrum disorder (ASD) (Myers et al., 2021). We included 26 studies that investigated monozygotic (MZ) twin pairs discordant for ASD and sought to explore biological mechanisms and phenotypic presentations associated with non‐shared environment. The review found that non‐shared environment effects in ASD might be associated with heterogeneous postzygotic genetic mechanisms and manifest as a range of biological and behavioral phenotypes. However, the depth of our findings was limited by relatively few studies, small sample sizes, and methodological diversity. We concluded that more research is needed on co‐occurring biological and behavioral phenotypes using a consistent format for designing, analyzing, and reporting MZ ASD discordant twin studies in order to further examine the role of non‐shared environment in the etiology of ASD. Reflecting on our systematic review, this editorial will provide an overview of twin design in research and its benefits and limitations. It will also describe areas in need of further rigorous research using twin design and suggests some standards related to twin design, analysis, and the publication of twin studies.

Sir Francis Galton is credited as one of the first to describe the classical twin design and in his book, *Inquiries into Human Faculty and its Development* (1883), he stated that “… twins have a special claim upon our attention; it is, that their history affords means of distinguishing between the effects of tendencies received at birth, and those that were imposed by the special circumstances of their after lives.” Nearly a century and a half later, twin research continues to be viewed as the via regia to explore the influences of genetics versus environment on a variety of biological mechanisms and behavioral traits. MZ twins are individuals who are derived from the same fertilized egg in utero and share nearly 100% of their genetics, while dizygotic (DZ) twins are derived from two separate fertilized eggs in utero and share roughly 50% of their genetics (Boomsma et al., 2002). This distinction allows researchers to compare traits in MZ versus DZ twins to explore the potential contribution of genetics versus environment in their research questions. With the twin design, the basic premise is that greater similarity on an outcome for MZ versus DZ twins may indicate strong genetic influence (Martin et al., 1997; Posthuma & Polderman, 2013).

There are several types of twin study designs that are available for research, including the classic twin design and the co‐twin control design, along with studies of twins who are adopted into different families. The designs may be cross‐sectional or longitudinal. Scurrah and Hopper (2019) and Boomsma et al. (2002) both provide helpful overviews of the different types of twin designs. In general, with twin studies, two assumptions are made: (i) equal environment and (ii) random assortative mating. Equal environment refers to the assumption that twins, regardless of zygosity, are raised in the same home. Therefore, no difference should exist in the effect of environment on MZ versus DZ twin pairs. A violation of equal environments may affect, for example, estimates of heritability. Assortative mating assumes there is random selection among the parents for the twins (Sahu & Prasuna, 2016; Willfors et al., 2017). A violation of the assortative mating assumption would mean parents procreate with individuals more phenotypically similar to themselves (e.g., both parents are tall). This may lead to DZ twins, in particular, who share more than 50% of their genomes (Sahu & Prasuna, 2016; Willfors et al., 2017). A variety of statistical methods can be applied to twin studies and are reviewed in Scurrah and Hopper (2019). These methods allow researchers to explore associations between genetics and environment with traits of interest, generally with some type of adjustment for the relatedness of the data (since the subjects are twins). Statistical methods also exist to explore heritability or causal inference using twin design in research.

Twin studies have several strengths and limitations. A primary strength is that most twins share a similar prenatal environment and some level of similarity in their upbringing (if they are raised together). Regardless of zygosity, twins are also the same age and generally have the same parents (although it is possible for DZ twins to have separate fathers). In contrast, sibling studies include offspring of different ages who subsequently may have different prenatal and postnatal environments. Siblings are also more likely than twins to have different fathers. Although twin studies can save time and costs compared with traditional case‐control studies where researchers need to find matched controls, there are some limitations. If one twin in the pair provides incomplete or unusable data for some reason, this may affect the researcher's ability to include the twin pair in statistical analyses. Another limitation to twin studies include the rarity of twins, especially those discordant for traits. There are also concerns whether or not being a twin places an individual at an increased risk for certain outcome and if results from twin studies can be generalized to non‐twin populations (Martin et al., 1997; Sahu & Prasuna, 2016; Scurrah & Hopper, 2019). For example, individuals who are twins may have lower birth weights or experience other complications in utero or in the early postnatal period, like prematurity; therefore, one can question if individuals who are twins are comparable to singletons (Buckler & Green, 2004).

In our research within the Roots of Autism and ADHD Twin Study Sweden (RATSS) (Bölte et al., 2014), we generally use the co‐twin control design (also known as discordant MZ pair design or within‐twin pair differences design) (Boomsma et al., 2002; Scurrah & Hopper, 2019). In the co‐twin control design, MZ twins discordant for an outcome of interest (in our case, neurodevelopmental conditions like ASD or attention‐deficit/hyperactivity disorder [ADHD]) are especially informative to our work as we attempt to uncover what was unique for one twin compared with the other that could have contributed or resulted in the neurodevelopmental outcome. Since RATSS began in 2011, we have recruited 226 pairs (includes two trios of triplets). In RATSS, the plan has always been to expand data collection longitudinally to reexamine previous participants, particularly those with greatest phenotypic discordance for neurodevelopmental diagnoses, symptoms, or traits. Previous longitudinal studies on twins exploring topics related to neurodevelopment have been able to further confirm or disconfirm relationships between outcomes of interest and genetic versus environmental influences on neurodevelopment (Christensen et al., 2021; Hallett et al., 2010; Taylor et al., 2015); however, these studies examined individuals over a relatively short time frame (e.g., 3–8 years). Longitudinal studies spanning longer periods of time are needed as individuals with neurodevelopmental conditions are now living longer than before and impact of these conditions on the aging process and quality of life has only minimally been explored to date (Anker et al., 2020). We also have expanded data the RATSS collection to include measures for neurodevelopmental and psychiatric conditions and biosamples (i.e., blood, saliva, hair) from biological parents and we collect a family pedigree. The extension of our study to include data from parents is an example of an extended twin family study where other family members, like biological parents or siblings, are included in the study to further explore genetic and environmental influences (Boomsma et al., 2002; Scurrah & Hopper, 2019). Using components of the extended family design, we published a case series exploring a rare genetic duplication in a MZ twin pair concordant for ASD and ADHD, along with their family members and other unrelated individuals with a similar duplication (Myers et al., 2020).

Although there is a growing body of research using the twin design to explore neurodevelopmental conditions (e.g., Curtin et al., 2022; Neufeld et al., 2021) more studies are needed that examine twins highly discordant for the outcome or trait(s) of interest (van Dongen et al., 2012), and to examine twins longitudinally over extended periods of time. Defining divergence (and even convergence) in twins is complicated as no universal standard exists for defining discordance in conditions like ASD, as outlined in our article (Myers et al., 2021) and definitions of discordance across studies can be quite varied (e.g., discordance in diagnosis, trait scores, symptoms, etc.) or sometimes not even fully described. Additionally, examining twins longitudinally, especially through the collection of biological and behavioral data over time, can be extremely valuable to future work in the area of neurodevelopmental conditions and other disorders that are considerable highly heritable. The use of twin registries worldwide that often collect data longitudinally over extended periods of time, such as those outlined in the article by van Dongen et al. (2012), coupled with the collection of biological samples, has the potential to allow for large‐scale molecular studies, for example,

However, twins remain rare and highly discordant twins for specific diagnoses, traits, or symptoms are even more rare. In RATSS, we have been able to collect one of the largest samples worldwide of twins qualitatively discordant for neurodevelopmental conditions. In RATSS, we use a consensus process with trained behavioral clinicians to diagnose the twin participants as neurotypical or having neurodevelopmental conditions like ASD or ADHD. We then use any diagnoses to determine the twin pair's qualitative concordance (both individuals in the twin pair have a diagnosis) or discordance (one individual in the twin pair has a diagnosis, while the other does not). Using qualitative discordance, we have found 25 MZ pairs (and 37 DZ pairs) discordant for ASD, 16 MZ (and 39 DZ) pairs discordant for ADHD, and 34 MZ pairs (and 44 DZ pairs) discordant for neurodevelopmental conditions (one individual in the twin pair has a neurodevelopmental condition, while the other does not). There are no clear standards on what defines quantitative discordance for neurodevelopmental conditions; however, some recent studies by our research group have used the cut‐off of the standard measurement of error on a validated tool for ASD traits (corresponding to seven points on the Social Responsiveness Scale‐2 questionnaire) as the criterion for quantitative discordance (Lundin Remnelius et al., 2022; Pan et al., 2020).

In our systematic review, a major challenge for our team when reviewing the available studies for inclusion was the lack of consistent and comprehensive reporting related to the design and analysis of twin studies. We were surprised to learn that—to the best of our knowledge—no known standards have ever been established for reporting the results of twin studies. While authors may use various reporting guidelines based on the type of study they perform (e.g., randomized trials, observational studies, etc.; see the Enhancing the QUAlity and Transparency Of health Research‐EQUATOR‐network website at equator‐network.org), the need to report special study characteristics related to the use of twins is critical.

In Table 1, we have compiled a list of recommended elements for studies on twins, with a focus on those exploring neurodevelopmental conditions in MZ discordant twins. The table includes minimal standards for the reporting on samples, methods, and analyses in twin studies, with the aim that authors provide sufficient and standardized information for readers that increase the value of published research on twin studies. We hope these proposed elements help further advance the science communication of environmental and genetic contributions to outcomes explored in MZ twin pair studies.

**TABLE 1:** Recommended elements for reporting studies on twins with neurodevelopmental conditions, including MZ discordant twins

Reporting element	Information to include	Information specific to MZ discordant twins
Samples	Sample recruitment method Any bias in recruitment or lack of representativeness of sample Demographics: number of twin pairs, twin pair breakdown by zygosity and concordance/discordance, ratio of female to male pairs, and breakdown of gender, race/ethnicity, age, age range, socioeconomic status, etc. by twin pair types	Demographics: number of MZ discordant twin pairs
Methods		
Diagnosis of Neurodevelopmental Conditions:	Use DSM or ICD (and version numbers) in diagnostic description Measures used for diagnosis or determination of traits Description of comorbidity and how handled	Cut‐off for dimensional discordance and/or discordance definition
Zygosity	Method for determination of zygosity (including validity and reliability of method)	
Blinding	Any blinding and how performed	
Drop‐out/missing data	Any incomplete data or drop‐outs of co‐twins Description of missing data and how handled	
Analysis	Statistical procedures account for paired nature of data/adjust for familial confounding factors List of outcome and predictor variables Statistical adjustments made (e.g., IQ)	Perform within pair analyses using MZ discordant pairs only

Abbreviation: MZ, monozygotic.

Although twin designs, including the discordant MZ pair design, have value in research on neurodevelopment and beyond, other designs are also worth noting, like sibling studies (using non‐twin full or half siblings) or adoption studies (either studies of twins adopted by different parents or an adopted sibling into a family) that allow researchers to further explore the effects of genetics and/or environment on outcomes of interest (Carlsson et al., 2020). Additionally, there is a need to replicate the findings from twin research in larger samples of non‐twins, such as what was done in Schwabe et al. (2017) who used results from twin studies on heritability of educational achievement and validated the results in a larger, census‐based sample. The use of these multiple methods to not only conduct, but also validate research findings, can help us further develop our understanding of conditions affecting neurodevelopment and beyond.

## AUTHOR CONTRIBUTIONS

**Lynnea Myers**: Conceptualization; Writing – original draft. **Sven Bölte**: Conceptualization; Supervision; Writing – review & editing.

## CONFLICT OF INTEREST

All authors declare no direct conflict of interest related to this editorial. S.B. discloses that he has in the last 3 years acted as an author, consultant, or lecturer for Medice and Roche. He receives royalties for textbooks and diagnostic tools from Hogrefe, Kohlhammer, and UTB.
